# Adverse birth outcomes in adolescent and young adult female cancer survivors: a nationwide population-based study

**DOI:** 10.1038/s41416-019-0712-2

**Published:** 2020-01-13

**Authors:** Wei-Heng Kao, Chang-Fu Kuo, Meng-Jiun Chiou, Yu-Cheng Liu, Chun-Chieh Wang, Ji-Hong Hong, Jun-Te Hsu, Yu-Jung Chiang, Yi-Fang Chuang

**Affiliations:** 1 Department of Radiation Oncology, Chang Gung Memorial Hospital, Taoyuan, Taiwan; 2 Institute of Public Health, National Yang-Ming University, Taipei, Taiwan; 3 Division of Rheumatology, Allergy and Immunology, Chang Gung Memorial Hospital, Taoyuan, Taiwan; 4 Division of Rheumatology, Orthopaedics and Dermatology, School of Medicine, University of Nottingham, Nottingham, UK; 5 Department of Gynecology and Obstetrics, Chang Gung Memorial Hospital, Taipei, Taiwan; 6 Department of Medical Imaging and Radiological Sciences, Chang Gung University, Taoyuan, Taiwan; 7 Radiation Biology Research Center, Institute for Radiological Research, Chang Gung University/Chang Gung Memorial Hospital, Taoyuan, Taiwan; 8 Department of General Surgery, Chang Gung Memorial Hospital, Taoyuan, Taiwan; 9 Department of Psychiatry, Far Eastern Memorial Hospital, Taipei, Taiwan

**Keywords:** Epidemiology, Cancer epidemiology

## Abstract

**Background:**

For female adolescent and young adult (AYA), cancer with treatments may affect their children’s health. Our aim was to determine reliable risk estimates of adverse birth outcomes in AYA cancer survivors and the differential effects of treatments.

**Methods:**

The study population of 4547 births in the AYA cancer survivor group and 45,463 in the comparison group were identified from two national databases between 2004 and 2014. Detailed maternal health conditions, such as maternal comorbidities, medication use during pregnancy and lifestyles, were adjusted in the statistical analyses. The outcomes included low birth weight, preterm labour, stillbirth, small or large for gestational age, a 5-min Apgar score <7, congenital malformation and foetal distress.

**Results:**

The AYA cancer survivor group had a 9% higher risk of overall adverse birth outcomes (adjusted odds ratio, 1.09; 95% confidence interval, 1.02–1.16), especially low birth weight and preterm labour than the comparison group. The radiotherapy-only group additionally had a higher risk of foetal distress, and a 5-min Apgar score <7.

**Conclusion:**

AYA cancer survivors, especially those who have received radiotherapy, still have higher risks of adverse birth outcomes after adjusting for detailed maternal health conditions. Preconception counselling and additional surveillance may be warranted in this population.

## Background

The incidence of cancer in adolescent and young adult (AYA) women aged 15 to 39 years^1^ has been gradually increasing in the United States and Europe.^2,3^ A similar condition exists in Taiwan, with ~60 new cancer cases per 100,000 people in 2000 rising to over 80 new cases per 100,000 people in 2015.^4^ However, with advances in cancer treatment and supportive care, the overall cancer survival rates at 5 years are over 80% and continue to increase.^5^ For some AYA female cancer survivors, having a child becomes a symbol of returning to normal life after cancer treatment. However, cancer-directed therapies may affect women’s cardiovascular functions,^6^ endocrine system,^7^ and fertility; even when fertility is preserved, worries about adverse birth outcomes may prevent female cancer survivors from attempting to conceive a child.

Scant information exists concerning birth outcomes in the AYA cancer survivors. Increased risks of preterm labour and low birth weights in AYA female cancer survivors have been observed.^8,9^ Similar results have been reported in cancer survivors of a wider (16–45 years) age range.^10,11^ Furthermore, results regarding the association between cancer treatments and adverse birth outcomes have been inconsistent. Anderson et al.^8^ reported that chemotherapy was associated with a higher risk of adverse birth outcomes, whereas Haggar et al.^9^ demonstrated that the same was true for radiotherapy (RT). The results might be inconsistent because most of these studies were based on cancer registry data with limited information regarding mothers’ health conditions, such as maternal comorbidities, medication use and lifestyle factors during pregnancy.^12–15^

In this population-based cohort study, two nationwide databases, those of the Taiwan National Health Insurance (NHI) and Taiwan Birth Reporting System (TBRS), were linked to obtain relevant information about maternal health conditions. The aim of this study was to estimate whether risks of adverse birth outcomes are higher in AYA cancer survivors than in the general population after rigorous adjustment for maternal health conditions. Furthermore, we investigated whether different cancer treatments, such as chemotherapy and radiation therapy, are associated with different adverse birth outcomes.

## Methods

### Data sources, study design and study population

This was a retrospective population-based cohort study that used the TBRS and NHI databases. In Taiwan, the TBRS was established in 1993, based on the “The Child Welfare Act”, which stipulates that medical organisations must submit a birth certificate to the health authority to facilitate the future health care for the mother and newborn. After submission, the staffs of the TBRS verify the information and correct possible errors. In 2004, the reporting system was fully converted to an online system with improved accuracy and completeness of information.^16^ Information collected by the TBRS includes adverse birth outcomes, maternal lifestyle data of alcohol misuse and smoking during pregnancy, and nationality.

The NHI database was established in 1996 and encompasses over 99.5% of the population of Taiwan. It contains comprehensive information on topics such as ambulatory and inpatient care, chronic mental illness care, medication and maternity care. The maternity care offered, including foetal ultrasound, delivery and postpartum and infant care, is fully covered by the NHI. The International Classification of Diseases, Ninth Revision codes are used for cancer and comorbidity diagnoses in the NHI database and are listed in Supplementary Table 1. Regarding the validity of cancer diagnoses in the NHI database, our previous study reported that the positive predictive value, negative predictive value, sensitivity and specificity were 94%, 100%, 91% and 100% for all cancers and positive predictive value ranged from 95% to 82% for the 10 leading cancer causes of death.^17^ For this study, we collected information on maternal covariates and cancer treatments from the NHI database.

**Fig. 1:**
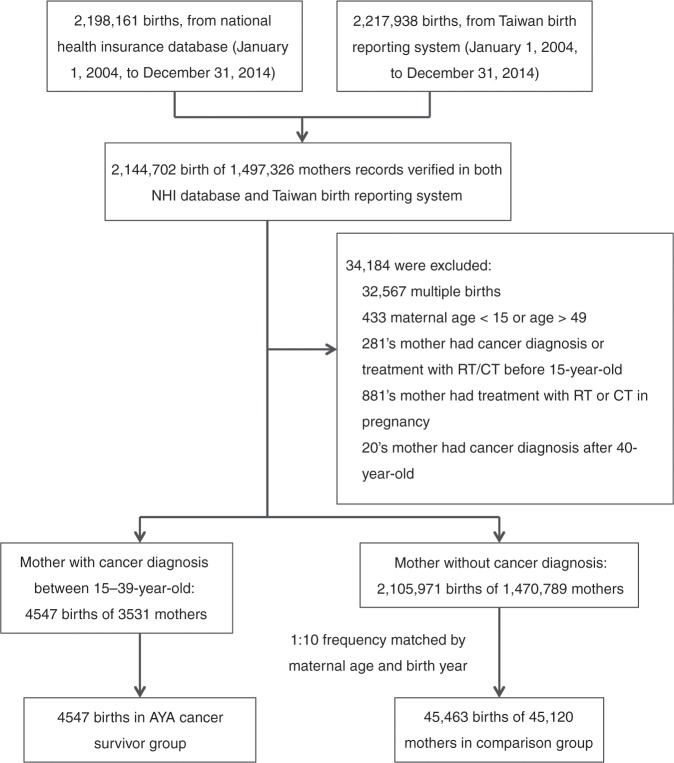
Flow chart.

First, we identified single births from 1 January 2004 to 31 December 2014 to mothers aged 15–49 years verified in both NHI and TBRS databases. A total of 2,144,702 births (1,497,326 mothers) were identified. Mothers who had a history of paediatric cancer, who had received RT/chemotherapy before the age of 15 years, or who had RT or chemotherapy during pregnancy (90 days before the date of the last menstrual period to birth) were then excluded to result in 2,110,518 births. Among them, 3531 mothers who were diagnosed with invasive cancer at the ages of 15–39 years and 4547 births after their cancer diagnoses were defined as the AYA cancer group. The comparison group, comprising 45,463 births to 45,120 mothers, was selected by randomly sampling 10 comparison births for every birth included in the AYA cancer group, using greedy (nearest-neighbour) matching algorithm for maternal age and infant birth year (Fig. 1).

Because of the complexity of procedure codes of surgeries for multiple cancer types, we categorised cancer treatments mainly based on the procedure codes of RT and medication codes of chemotherapies. For AYA cancer survivors who received neither chemotherapy nor RT, we assumed they only had undergone surgery. A total of 67% (*n* = 3045) received neither RT nor chemotherapy, 1.9% (*n* = 84) received RT alone, 0.2% (*n* = 10) received chemotherapy alone, and 31.0% (*n* = 1408) received chemotherapy plus RT. In cancer treatment analysis, we combined the chemotherapy-alone group with the chemotherapy plus RT group (as the chemotherapy with or without RT group) because of the small number of people within the chemotherapy-alone group.

### Adverse birth outcomes

Adverse birth outcomes from the TBRS data comprised stillbirth, low birth weight (<2500 g), preterm labour (<37 weeks), small for gestational age (birth weight below the 10th percentile for gestational age), large for gestation age (birth weight above the 90th percentile for gestational age), 5-min Apgar score <7, congenital malformation and foetal distress. Being small or large for gestational age was based on a nomogram of all live births from 2004 through 2014 in the TBRS database. In addition, caesarean delivery (both elective and non-elective) was included as a birth outcome.

### Covariates

We obtained demographic and socioeconomic information on the date of delivery, including maternal age at delivery, nationality, place of residence, income level, and occupation, from the NHI database. Maternal place of residence was categorised as urban, suburban or rural according to the level of urbanisation of the 369 towns in Taiwan.^18^ Income level was estimated and divided among the sex-specific quintiles in Taiwan. Maternal comorbidities of cardiovascular disease, autoimmune disease, liver disease,^19^ hypertension, diabetes mellitus (DM) before pregnancy, and gestational DM were also obtained from the NHI database. These have been well validated, with excellent results.^20–22^ For maternal medication use during pregnancy, we included selective serotonin reuptake inhibitors, and pregnancy category D and X medications.^23^ The relevant time period for medication use during pregnancy was defined as from 90 days before the date of the last menstrual period to the birth date. The detailed codes of comorbidities and medications are listed in Supplementary Tables 1 and 2. Otherwise, maternal lifestyle factors, comprising alcohol misuse and smoking during pregnancy as well as infant sex and birth year, were obtained from the TBRS database.

### Statistical analysis

Differences in demographic and socioeconomic variables between the AYA cancer survivor group and the comparison group were assessed using *χ*^2^ tests. Because mothers may have given consecutive births, a generalised estimating equation model was used to estimate the odds ratio (OR) with a 95% confidence interval (CI) for each birth outcome comparing the AYA cancer survivor group and the comparison group. The correlation structure was based on an autoregressive model. First, the basic model included maternal demographics, maternal lifestyle factors, infant sex and birth year. The full model was further adjusted for maternal comorbidities and medication use during pregnancy and each comorbidities and medication types were presented as a binary (yes/no) variable. The associations between adverse birth outcomes and cancer treatments were examined using the full model. We also performed a sensitivity analysis to estimate the risks of adverse birth outcomes among cancer survivors with a wider age range for cancer diagnosis (15–49 years). In order to clarify the impact of cancer types with different cancer treatments, we performed sensitivity analyses in thyroid, gynaecologic and breast cancer survivors separately. In addition, the associations within different groups of cancer diagnosis age (<30 and ≥30 years) and different length of duration between cancer diagnosis and delivery (<3 years and ≥3 years) were also examined. A two-tailed test with a 5% level of significance was used for all statistical hypotheses. All analyses were conducted using the SAS software, v.9.4 (SAS Institute, Cary, NC).

## Results

Characteristics of the study population are listed in Table 1. The most common cancer diagnosis among cancer survivors was thyroid cancer (*n* = 1584 [34.8%]), followed by gynaecologic cancer (*n* = 725 [15.9%]) and breast cancer (*n* = 691 [15.2%]). The median age of cancer diagnosis was 27.1 years, and the median time between cancer diagnosis and delivery was 4.6 years. The median age at delivery was 33.0 years. The AYA cancer survivors tended to have more medical comorbidities than the comparison group. No difference between the two groups was observed in medication use during pregnancy. In the RT-alone group, the main cancer types were gynaecologic cancer (23%), breast cancer (18%) and thyroid cancer (15%). The main cancer types in the chemotherapy group with or without RT were breast cancer (36%), gynaecologic cancer (16%) and non-Hodgkin’s lymphoma (9%).

**Table 1:** Characteristics between adolescent and young adult cancer survivor group and comparison group.

Characteristics	No. (%) of births		
	Comparison group *N* = 45,463 to 45,120 mothers	Cancer survivor group *N* = 4547 to 3531 mothers	*P* value
Maternal age at delivery			>0.99
15–24 years	1480 (3.3)	148 (3.3)	
25–34 years	28,820 (63.4)	2882 (63.4)	
≥35 years (max. 48 years)	15,163 (33.4)	1517 (33.4)	
Median	33.0 years	33.0 years	
Infant gender			0.355
Female	21,680 (47.7)	2201 (48.4)	
Infant birth year			>0.99
2004–2007	12,712 (28.0)	1272 (28.0)	
2008–2011	16,122 (35.5)	1612 (35.5)	
2012–2014	16,629 (36.6)	1663 (36.5)	
Maternal comorbidity			
Cardiovascular disease	290 (0.6)	62 (1.4)	<0.001
Autoimmune disease	507 (1.1)	72 (1.6)	0.007
Liver disease	669 (1.5)	205 (4.5)	<0.001
Hypertension	656 (1.3)	92 (2.0)	0.003
Diabetes mellitus before pregnancy	768 (1.7)	103 (2.27)	0.007
Gestational diabetes mellitus	1469 (3.0)	166 (3.7)	0.136
Maternal lifestyle			
Smoking during pregnancy	41 (0.1)	–^a^	0.267
Alcohol misuse during pregnancy	8 (0.0)	–^a^	0.217
Maternal selective serotonin reuptake inhibitor use during pregnancy^b^	270 (0.6)	25 (0.6)	0.708
Maternal category D and X medication use during pregnancy^b^	53 (0.1)	4 (0.1)	0.571
Maternal cancer types			
Thyroid cancer		1584 (34.8)	
Gynaecologic cancer		725 (15.9)	
Breast cancer		691 (15.2)	
Others		1547 (34.0)	
Maternal cancer treatments			
No CT or RT	–	3045 (67.0)	
RT alone	–	84 (1.9)	
CT with or without RT	–	1418 (31.2)	
Maternal nationality			<0.001
Taiwan	42,698 (93.9)	4465 (98.2)	
Maternal place of residence			<0.001
Urban	39,031 (85.9)	4002 (88.0)	
Suburban	5433 (12.0)	471 (10.4)	
Rural	999 (2.2)	74 (1.6)	
Maternal income levels (New Taiwan Dollar)^c^			<0.001
Quintile 1 (≤21,900)	4983 (11.0)	375 (8.25)	
Quintile 2 (22,800–28,000)	10,588 (23.3)	1123 (24.7)	
Quintile 3 (30,300–40,100)	9767 (21.5)	941 (20.7)	
Quintile 4 (42,000–53,000)	8677 (19.1)	912 (20.1)	
Quintile 5 (≥55,400)	11,448 (25.2)	1196 (26.3)	
Maternal occupation			<0.001
Dependents of the insured individuals	26,424 (58.12)	2693 (59.23)	
Civil servants, teachers, military personnel and veterans	2144 (4.72)	254 (5.59)	
Non-manual workers and professionals	14,702 (32.34)	1343 (29.54)	
Manual workers/others	2193 (4.82)	257 (5.65)	

^a^Numbers <3 are not displayed, as per the confidentiality policies of National Health Insurance Database

^b^From 90 days before the date of the last menstrual period to the birth date

^c^Maternal income levels were estimated by insurance premium using sex-specific quintiles in Taiwan from low to high

Table 2 presents the results on the risks of adverse birth outcomes of the AYA cancer survivor group and comparison group. The prevalence of all adverse birth outcomes (excluding caesarean delivery) increased from 34% in the comparison group to 36% in the cancer survivor group, indicating a 9% higher overall risk of adverse birth outcomes in the AYA cancer survivor group (OR, 1.09; 95% CI, 1.02–1.16). Among them, significantly higher risks of low birth weight (OR, 1.15; 95% CI, 1.02–1.30) and preterm labour (OR, 1.12; 95% CI, 1.00–1.25) were found. In addition, the AYA cancer survivors tended to require a caesarean delivery (OR, 1.18; 95% CI, 1.10–1.27). The risk estimates did not change significantly with further adjustment for maternal comorbidities and medication use during pregnancy.

**Table 2:** Adverse birth outcomes of infants in the female adolescent and young adult (AYA) cancer survivor group and the comparison group.

	Comparison group *N* = 45,463	AYA cancer survivor group *N* = 4567	Crude odds ratio (95% CI)	Adjusted odds ratio^a^ Model 1 (95% CI)	Adjusted odds ratio^b^ Model 2 (95% CI)
	*N* (%)	*N* (%)			
Overall adverse birth outcome^c^	15,436 (33.95)	1643 (36.13)	1.1 (1.03–1.18)	1.1 (1.03–1.18)	1.09 (1.02–1.16)
Stillbirth	391 (0.86)	41 (0.90)	1.05 (0.76–1.45)	1.04 (0.75–1.43)	1.01 (0.74–1.40)
Low birth weight	3121 (6.86)	366 (8.05)	1.19 (1.06–1.34)	1.18 (1.05–1.32)	1.15 (1.02–1.30)
Preterm labour	3788 (8.33)	439 (9.65)	1.16 (1.04–1.29)	1.15 (1.04–1.29)	1.12 (1.00–1.25)
Small for gestational age	4109 (9.04)	436 (9.59)	1.08 (0.97–1.20)	1.07 (0.96–1.19)	1.07 (0.96–1.19)
Large for gestational age	4965 (10.92)	511 (11.24)	1.03 (0.93–1.14)	1.04 (0.94–1.16)	1.03 (0.93–1.14)
5-min Apgar score <7	480 (1.06)	57 (1.25)	1.19 (0.90–1.57)	1.19 (0.90–1.57)	1.14 (0.86–1.51)
Congenital malformation	2817 (6.20)	289 (6.36)	1.03 (0.91–1.18)	1.01 (0.89–1.15)	1.01 (0.89–1.15)
Foetal distress	2205 (4.85)	250 (5.50)	1.14 (0.99–1.31)	1.14 (0.99–1.31)	1.14 (0.99–1.31)
Caesarean delivery	17,297 (38.05)	1889 (41.54)	1.2 (1.12–1.28)	1.19 (1.11–1.27)	1.18 (1.10–1.27)

^a^Model 1: Maternal age at delivery, infant sex, infant birth year, maternal alcohol misuse during pregnancy, maternal smoking during pregnancy, maternal nationality, maternal place of residence, maternal income level, maternal occupation

^b^Model 2: Model 1 + maternal cardiovascular disease, maternal autoimmune disease, maternal liver disease, maternal hypertension, maternal diabetes mellitus before pregnancy, maternal gestational diabetes mellitus, maternal bipolar disorder, maternal selective serotonin reuptake inhibitor use during pregnancy, maternal category D and X medication use during pregnancy

^c^Caesarean delivery excluded

The risks of adverse birth outcomes associated with different cancer treatments are presented in Table 3. The adverse birth outcome rate in the neither RT nor chemotherapy group was comparable to the comparison group. The RT-alone group had the highest risk of overall adverse birth outcomes (OR, 2.42; 95% CI, 1.52–3.85). Multiple adverse birth outcomes, including low birth weight (OR, 2.25; 95% CI, 1.20–4.23), preterm labour (OR, 2.40; 95% CI, 1.28–4.49), 5-min Apgar score <7 (OR, 3.63; 95% CI, 1.20–10.99) and foetal distress (OR, 2.79; 95% CI, 1.42–5.45), were significantly higher in the RT-alone group. In the chemotherapy with or without RT group, only the risk of low birth weight was significantly higher (OR, 1.23; 95% CI, 1.01–1.50). Finally, the survivors in all three treatment groups had higher caesarean delivery rates.

**Table 3:** Adverse birth outcomes in different anticancer treatment groups and the comparison group.

	Comparison group *N* = 45,463	Cancer survivor group					
		Neither RT nor chemotherapy *N* = 3045	Adjusted odds ratio^a^ (95% CI)	RT alone *N* = 84	Adjusted odds ratio^a^ (95% CI)	Chemotherapy ± RT *N* = 1418	Adjusted odds ratio^a^ (95% CI)
	*N* (%)	*N* (%)		*N* (%)		*N* (%)	
Overall adverse birth outcome^b^	15,436 (33.95)	1079 (35.44)	1.06 (0.98–1.15)	46 (54.76)	2.42 (1.52–3.85)	518 (36.53)	1.09 (0.98–1.22)
Stillbirth	391 (0.86)	26 (0.85)	0.97 (0.65–1.45)	0 (0)	NA	15 (1.06)	1.16 (0.70–1.93)
Low birth weight	3121 (6.86)	229 (7.52)	1.08 (0.94–1.26)	12 (14.29)	2.25 (1.20–4.23)	125 (8.82)	1.23 (1.01–1.50)
Preterm labour	3788 (8.33)	280 (9.20)	1.07 (0.93.22)	16 (19.05)	2.40 (1.28–4.49)	143 (10.08)	1.17 (0.97–1.40)
Small for gestational age	4109 (9.04)	298 (9.79)	1.10 (0.97–1.25)	10 (11.90)	1.53 (0.79–2.94)	128 (9.03)	0.98 (0.81–1.19)
Large for gestational age	4965 (10.92)	335 (11.00)	1.01 (0.89–1.14)	12 (14.29)	1.34 (0.71–2.54)	164 (11.57)	1.06 (0.89–1.26)
5-min Apgar score of <7	480 (1.06)	35 (1.15)	1.09 (0.76–1.55)	–^c^	3.63 (1.20–10.99)	19 (1.34)	1.12 (0.70–1.77)
Congenital malformation	2817 (6.20)	177 (5.81)	0.94 (0.80–1.10)	8 (9.52)	1.66 (0.77–3.59)	104 (7.33)	1.13 (0.91–1.39)
Foetal distress	2205 (4.85)	167 (5.48)	1.13 (0.96–1.34)	10 (11.90)	2.79 (1.42–5.45)	73 (5.15)	1.06 (0.83–1.36)
Caesarean delivery	17,297 (38.05)	1225 (40.23)	1.13 (1.04–1.22)	48 (57.14)	2.02 (1.32–3.09)	616 (43.44)	1.26 (1.11–1.42)

^a^Adjusted for maternal age at delivery, infant sex, infant birth year, maternal cardiovascular disease, maternal autoimmune disease, maternal liver disease, maternal hypertension, maternal diabetes mellitus before pregnancy, maternal gestational diabetes mellitus, maternal bipolar disorder, maternal alcohol misuse during pregnancy, maternal smoking during pregnancy, maternal selective serotonin reuptake inhibitor use during pregnancy, maternal category D and X medication use during pregnancy, maternal nationality, maternal place of residence, maternal income level, maternal occupation

^b^Caesarean delivery excluded

^c^Numbers <3 are not displayed, as per the confidentiality policies of National Health Insurance Database

For the sensitivity analysis with the wider age group (15–49 years), similar results were observed, with a higher overall risk of adverse birth outcomes, especially low birth weight and preterm labour (Supplementary Tables 3 and 4). Higher rates of preterm labour, foetal distress and 5-min Apgar score <7 were also noted in the RT-alone group. The risks of low birth weight were also high in both the RT-only group and the chemotherapy with or without RT group. In the analysis of the thyroid, gynaecologic and breast cancer survivors with different cancer treatments, higher preterm labour and low birth weight were noted in both thyroid and gynaecologic cancer survivors in the RT-alone group (Supplementary Table 5, 6 and 7). For cancer survivors who had diagnosis before and above 30 years old, both of them had higher risks of overall risk of adverse outcomes in the RT-alone group (Supplementary Table 8). The duration between cancer diagnosis and delivery (<3 and ≥3 years) showed similar risks in neither RT nor chemotherapy group and chemotherapy with and without RT group. Only in RT-alone group, lower risks of overall adverse birth outcome were noted when their duration over 3 years (Supplementary Table 9).

## Discussion

In this population-based study of over 2 million births in Taiwan, we observed that AYA female cancer survivors had a 9% higher risk of overall adverse birth outcomes compared with the comparison group after rigorous adjustment for maternal comorbidities, medication use during pregnancy, and lifestyle factors. Particularly, the risks of preterm labour and low birth weight significantly increased in the cancer survivors’ group. Furthermore, among different cancer treatments, cancer survivors receiving RT alone had a 2-fold increase in the risk of overall adverse birth outcomes, including low birth weight, preterm labour, foetal distress and Apgar score <7 at 5 min.

Our findings regarding preterm labour and low birth weight are in agreement with those of similar studies in western countries. Anderson et al.^8^ reported a 1.52-fold preterm labour risk and a 1.59-fold lower birth weight risk in infants of AYA cancer survivors in the United States. Furthermore, similar results (1.5- to 2-fold increase) in risks of preterm labour and low birth weight were reported by Haggar et al.^9^ in infants of AYA cancer survivors in Western Australia and by Stensheim et al.^11^ in infants of female cancer survivors aged 16–45 years in Norway. In the studies where infants of childhood and AYA cancer survivors were combined, higher risk of preterm delivery and low birth weight were also reported.^24,25^ Later in the meta-analysis, which included both childhood and AYA female cancer survivors, a 1.5-fold risk increase of preterm labour and low birth weight were summarised.^26^ However, in our research, we observed a smaller magnitude of the increased risk in preterm labour risk (12%) and low birth weight (15%). A possible explanation is the different prevailing cancer types in the AYA cancer survivors’ group in our study (thyroid cancer, breast cancer and ovarian cancer) from those of other western country studies (melanoma, thyroid cancer and breast cancer). This difference may cause different proportions of cancer treatments, such as RT or chemotherapy. A higher prevalence of certain cancer types that require these treatments may result in a higher magnitude of risks. The other explanation is that we may have adjusted more potential confounders regarding maternal medication use and lifestyle factors during pregnancy as well as maternal health conditions.

The toxicities to female reproductive organs are mixed by both RT and chemotherapy. Our findings suggest that RT and chemotherapy may cause a higher risk of adverse birth outcomes, especially preterm labour and low birth weight; this result is consistent with Haggar et al.^9^ and Anderson et al.^8^ In the meta-analysis by van der Kooi et al.^26^, 2.3-fold risk of preterm labour was similar to our results in the RT-alone group, although their result was mainly based on childhood cancer survivors (three of four studies). The possible mechanisms underlying the associations between adverse birth outcomes and RT and chemotherapy may be related to the toxicities of these treatments to the ovaries. In a study of childhood cancer survivors, RT applied to a female’s abdomen was significantly associated with low birth weight and preterm labour.^27^ Radiation damages include direct damage, double-stand breaks and indirect damage, and free radical formation. When organs develop and cells proliferate, they become more sensitive to these damages. RT can result in a low median lethal dose of 2 Gy to the ovaries, which is 3–10% of a regular treatment dose.^28^ Radiation also damages the endometrium, myometrium and regional vascular uterine structures and functions that are essential for foetal development.^29,30^ For chemotherapy toxicities, high-dose alkylating agents or platinum-based compounds may cause cross-links during replication and P63-mediated apoptotic death in human primordial follicles.^31^

In our analysis of cancer types, both thyroid and gynaecologic cancer survivors who received RT wound have higher risks of preterm labour and low birth weight, whereas in breast cancer survivors, we did not show increased risk of any adverse birth outcomes. However, higher rates of preterm labour and low birth weight were reported in female breast cancer survivors in previous studies without detailed information of RT or chemotherapy.^10,32,33^ The possible explanation might be that the radiation still bears the risk to scatter to reproductive organs even the radiation is not directly applied at the pelvis. Therefore, when AYA female cancer patients require RT, proper radiation protection by a lead shield over the pelvic region should be considered. It is still unclear how the duration between cancer diagnosis and giving birth to a child would influence adverse birth outcomes. Anderson et al.^8^ reported that there were no differences in risks of adverse birth outcomes with different durations up to 5 years, while Black et al.^33^ suggested that there was a decreased risk at giving birth to a child over 2 years after cancer diagnosis. In our findings, for cancer survivors who received RT alone and duration between cancer diagnosis and delivery <3 years had higher risks of adverse birth outcomes.

Strengths of this nationwide population-based study include the large number of births to AYA female cancer survivors and more abundant information about maternal lifestyle factors, medication use during pregnancy and maternal comorbidity from the health insurance database rather than could be obtained from a cancer registry. However, some limitations require consideration. First, there is a possibility of chance findings because we investigated multiple outcomes and a relatively small number of birth outcome events. However, our analysis of a wider cancer-diagnosed age group (15–49 years) with larger number of birth outcome events still presented similar results. Second, because the records of cancer diagnoses in the NHI database could only be traced back to 1996, the female adult (≥15 years) cancer survivors whose cancer was diagnosed before 1996 may have been misclassified. Theoretically, it is a nondifferential misclassification and leads the bias toward the null. Third, about the congenital malformation of adverse birth outcomes, we only included the instant findings at birth recorded in TBRS and might miss some minor congenital malformations discovered later. In other words, we might have underestimated the risk of congenital malformation. Finally, about the cancer treatments, we do not have the detailed information of RT treatment field, total treatment dose and dose distribution of organ at risk. In the meantime, due to lack of individual weight and body surface area, we had no information of chemotherapy dose in plasma and total cumulative dose.

## Conclusions

AYA cancer survivors were observed to have higher risks of overall adverse birth outcomes, especially preterm labour and low birth weight. For survivors who had received RT or chemotherapy, significantly higher risks of multiple adverse outcomes were found. Overall, our findings suggest preconception counselling, careful prenatal care and additional surveillance for female AYA cancer survivors are warranted.

## Supplementary information

Supplementary table

## Data Availability

The data sources are the Taiwan Nation Health Insurance Database and TBRS. The data that support the findings of this study are available from Taiwan Health and Welfare Data Science Centre. Restrictions apply to the availability of these data, which were used under license for this study. Data are available at https://dep.mohw.gov.tw/DOS/np-2497-113.html with the permission of Taiwan Health and Welfare Data Science Centre.
