# Supplementary material for: Adverse birth outcomes in adolescent and young adult female cancer survivors: a nationwide population-based study
Source: Br J Cancer. 2020 Jan 13;122(6):918–24. doi: 10.1038/s41416-019-0712-2 (PMC7078184; doi:10.1038/s41416-019-0712-2)
Supplement: Supplementary file 1 — Supplementary table [file 41416_2019_712_MOESM1_ESM.docx]

Supplementary table 1 International Classification of Diseases, 9th revision (ICD-9) codes for cancer and comorbidities diagnoses

| Deliver | V27, 650, 651, 652 |
| --- | --- |
| Cancer | 140-208 |
| Cardiovascular disease | 410.x, 412.x, 398.91, 402.01, 402.11, 402.91,404.01, 404.03, 404.11, 404.13, 404.91, 404.93, 425.4–425.9, 428.x, 093.0, 437.3, 440.x, 441.x, 443.1–443.9, 47.1, 557.1, 557.9, V43.4, 362.34, 430.x–438.x |
| Autoimmune disease | 446.5, 710.0–710.4, 714.0– 714.2, 714.8, 725.x |
| Liver disease | 070.22, 070.23, 070.32, 070.33, 070.44, 070.54, 070.6, 070.9, 570.x, 571.x, 573.3, 573.4, 573.8, 573.9  456.0–456.2, 572.2–572.8 |
| Hypertension | 401.x, 402.x, 403.x, 404, 405, 642 |
| Diabetes mellitus | 250.xx |
| Gestation diabetes mellitus | 648.8 |

Supplement table 2 Codes of selective serotonin reuptake inhibitors (SSRIs) and pregnancy category D and X medication

| Selective serotonin reuptake inhibitors | | |
| --- | --- | --- |
| CITALOPRAM HYDROBROMIDE | ESCITALOPRAM | FLUOXETINE |
| FLUVOXAMINE MALEATE | PAROXETINE HYDROCHLORIDE | SERTRALINE HYDROCHLORIDE |

| Pregnancy category D and X medication | | | | |
| --- | --- | --- | --- | --- |
| abiraterone | acitretin | actinomycin D | adapalene | aflibercept |
| albendazole | aliskiren | altretamine | ambrisentan | amikacin |
| aminoglutethimide | aminoglutethimide | amlodipine | amsacrine | arsenic trioxide |
| artemether with lumefantine | asparaginase | atorvastatin | axitinib | azacitidine |
| azathioprine | basiliximab | bendamustine hydrochloride | benzoyl peroxide | bevacizumab |
| bicalutamide | bleomycin | boceprevir | bosentan | brentuximab vedotin |
| busulfan | cabazitaxel | candesartan cilexetil | capecitabine | captopril |
| carbamazepine | carboplatin | carboprost | carmustine | ceritinib |
| cerivastatin | cetrorelix | cetuximab | chlorambucil | chloroquine |
| cidofovir | cilazapril | cisplatin | cladribine | clindamycin |
| clofarabine | cobimetinib fumarate | colaspase (asparaginase) | colchicine | conjugated estrogens |
| crizotinib | cyclophosphamide | cyproterone acetate | cytarabine | dabrafenib mesilate |
| dacarbazine | dactinomycin | danazol | dapagliflozin | dasatinib |
| daunorubicin | deferiprone | degarelix | demeclocycline | denosumab |
| dienoestrol | docetaxel | dofetilide | doxorubicin | doxycycline |
| dronedarone | dutasteride | efavirenz | empagliflozin | dronedarone |
| enalapril | enzalutamide | epirubicin | eprosartan | eptotermin alfa |
| eribulin | estramustine | etanercept | ethosuximide | etoposide |
|  |  |  |  |  |
| etretinate | finasteride | fingolimod | fluconazole | fludarabine |
| fluorouracil | fluoxymesterone | fluvastatin | follitropin alfa | fosinopril |
| fotemustine | framycetin | fulvestrant | ganciclovir | ganirelix |
| gemcitabine | gentamicin | gestrinone | goserelin | hydroxycarbamide |
| hydroxychloroquine | hydroxyprogesterone | hydroxyurea | ibrutinib | idarubicin |
| idelalisib | ifosfamide | imatinib | interferon beta-1a | interferon beta-1b |
| iodoform with bismuth subnitrate | irbesartan | irinotecan | isotretinoin | ivabradine |
| kanamycin | lamotrigine | ledipasvir | leflunomide | lenalidomide |
| lenvatinib | letrozole | leuprorelin | lisinopril | lithium salts |
| lomustine | losartan | macitentan | medroxyprogesterone | megestrol |
| melphalan | mercaptopurine | mesterolone | methacycline | methenolone |
| methotrexate | methsuximide | methylene blue | methylphenobarbital | methyltestosterone |
| miglustat | minocycline | misoprostol | mitomycin | mitozantrone |
| mustine | mycophenolate mofetil | mycophenolic acid | nafarelin | nandrolone |
| neomycin | netilmicin | nicotine | nilotinib | nintedanib esilate |
| nivolumab | norethisterone | olaparib | olmesartan | osimertinib |
| oxaliplatin | oxandrolone | oxcarbazepine | oxybuprocaine | oxymetholone |
| oxytetracycline | paclitaxel | panobinostat | paraldehyde | paritaprevir |
| paroxetine | pazopanib | peginterferon beta-1a | pembrolizumab | pemetrexed |
| penicillamine | penicillamine | perindopril | pertuzumab | phenindione |
| phenobarbital | phensuximide | phenytoin sodium | pitavastatin | plerixafor |
| podophyllotoxin | pomalidomide | ponatinib | pravastatin | primaquine phosphate |
| primidone | procarbazine | quinapril | quinine | radium (223Ra) dichloride |
| raloxifene | raltitrexed | ramipril | ramucirumab | ranibizumab |
| regorafenib | ribavirin | riociguat | romidepsin | rosuvastatin |
| samarium | simeprevir | simvastatin | sitaxentan | smallpox (Vaccinia) vaccine |
| sodium phosphate [32P] | sodium valproate | sofosbuvir | sorafenib | sulthiame |
| sunitinib | tasonermin | tazarotene | tegafur | telmisartan |
| temozolomide | temsirolimus | teniposide | teriflunomide | terlipressin |
| testosterone | tetracosactide | tetracosactrin | tetracycline | thalidomide |
| thiotepa | tibolone | tigecycline | tioguanine | tobramycin |
| tofacitinib | tolvaptan | topiramate | topotecan | trametinib dimethyl sulfoxide |
| trandolapril | trastuzumab emtansine (rch) | tretinoin | triptorelin | valganciclovir |
| valsartan | vemurafenib | vigabatrin | vinblastine | vincristine |
| vindesine | vinflunine | vinorelbine tartrate | vismodegib | vitamin A |
| vorinostat | warfarin | zalcitabine | zonisamide |  |

Supplement table 3 Adverse birth outcomes of infants in female cancer survivor group (diagnosed at age 15-49 years) and the comparison group

|  | Comparison group  N=45,662 | | Cancer survivor group  N=4,567 | | Crude odds ratio  (95% CI) | | Adjusted odds ratio^a^  Model 1 (95% CI) | | Adjusted odds ratio^b^  Model 2 (95% CI) | |
| --- | --- | --- | --- | --- | --- | --- | --- | --- | --- | --- |
|  | N | % | N | % |  |  |  |  |  |  |
| Overall adverse birth outcome^c^ | 15,511 | (33.97) | 1,654 | (36.22) | 1.11 | (1.04- 1.18) | 1.1 | (1.03- 1.18) | 1.09 | (1.02- 1.17) |
| Stillbirth | 398 | (0.87) | 41 | (0.90) | 1.03 | (0.75- 1.42) | 1.02 | (0.74- 1.40) | 1 | (0.72- 1.37) |
| Low birth weight | 3,140 | (6.88) | 367 | (8.04) | 1.19 | (1.06- 1.34) | 1.17 | (1.04- 1.32) | 1.15 | (1.02- 1.29) |
| Preterm labor | 3,812 | (8.35) | 442 | (9.68) | 1.16 | (1.04- 1.29) | 1.16 | (1.04- 1.29) | 1.12 | (1.01- 1.25) |
| Small for gestational age | 4,128 | (9.04) | 438 | (9.59) | 1.08 | (0.97- 1.20) | 1.07 | (0.96- 1.19) | 1.07 | (0.96- 1.19) |
| Large for gestational age | 4,992 | (10.93) | 517 | (11.32) | 1.04 | (0.94- 1.15) | 1.05 | (0.95- 1.16) | 1.04 | (0.94- 1.15) |
| 5-minute Apgar Score of < 7 | 489 | (1.07) | 57 | (1.25) | 1.17 | (0.88- 1.54) | 1.16 | (0.88- 1.54) | 1.12 | (0.84- 1.48) |
| Congenital malformation | 2,830 | (6.20) | 290 | (6.35) | 1.03 | (0.91- 1.17) | 1.01 | (0.89- 1.15) | 1.01 | (0.89- 1.15) |
| Fetal distress | 2,213 | (4.85) | 251 | (5.50) | 1.14 | (0.99- 1.31) | 1.14 | (0.99- 1.31) | 1.14 | (0.99- 1.31) |
| Cesarean delivery | 17,414 | (38.14) | 1,904 | (41.69) | 1.20 | (1.12- 1.29) | 1.19 | (1.11- 1.27) | 1.18 | (1.10- 1.27) |

^a^ Model 1: maternal age at delivery, infant sec, infant birth year, maternal alcohol misuse during pregnancy, maternal smoking during pregnancy, maternal nationality, maternal place of residence, maternal income level, maternal occupation

^b^ Model 2: Model 1+ maternal cardiovascular disease, maternal autoimmune disease, maternal liver disease, maternal hypertension, maternal diabetes mellitus before pregnancy, maternal gestational diabetes mellitus, maternal bipolar disorder, maternal selective serotonin reuptake inhibitor use during pregnancy, maternal category D and X medication use during pregnancy

^c^ Cesarean delivery excluded

|  | Comparison group  N=45,662 | | Cancer survivor group | | | | | | | | | | | |
| --- | --- | --- | --- | --- | --- | --- | --- | --- | --- | --- | --- | --- | --- | --- |
|  |  |  | Neither RT nor chemotherapy  N=3,059 | | Adjusted odds ratio^a^  Model 2 (95% CI) | | RT alone  N=84 | | Adjusted odds ratio^a^  Model 2 (95% CI) | | Chemotherapy ± RT  N=1,424 | | Adjusted odds ratio^a^  Model 2 (95% CI) | |
|  | N | % | N | % |  |  | N | % |  |  | N | % |  |  |
| Overall adverse birth outcome^b^ | 15,511 | (33.97) | 1,087 | (35.53) | 1.07 | (0.99- 1.16) | 46 | (54.76) | 2.42 | (1.52- 3.86) | 521 | (36.59) | 1.1 | (0.98- 1.23) |
| Stillbirth | 398 | (0.87) | 26 | (0.85) | 0.96 | (0.64- 1.43) | 0 | (0.00) | N/A | | 15 | (1.05) | 1.14 | (0.68- 1.89) |
| Low birth weight | 3,140 | (6.88) | 229 | (7.49) | 1.08 | (0.93- 1.25) | 12 | (14.29) | 2.25 | (1.20- 4.22) | 126 | (8.85) | 1.23 | (1.02- 1.50) |
| Preterm labor | 3,812 | (8.35) | 283 | (9.25) | 1.07 | (0.94- 1.23) | 16 | (19.05) | 2.4 | (1.28- 4.50) | 143 | (10.04) | 1.16 | (0.96- 1.39) |
| Small for gestational age | 4,128 | (9.04) | 299 | (9.77) | 1.1 | (0.97- 1.25) | 10 | (11.90) | 1.53 | (0.79- 2.94) | 129 | (9.06) | 0.99 | (0.82- 1.19) |
| Large for gestational age | 4,992 | (10.93) | 339 | (11.08) | 1.02 | (0.90- 1.15) | 12 | (14.29) | 1.34 | (0.71- 2.55) | 166 | (11.66) | 1.07 | (0.90- 1.27) |
| 5-minute Apgar Score of < 7 | 489 | (1.07) | 35 | (1.14) | 1.07 | (0.75- 1.52) | -* |  | 3.61 | (1.19- 10.93) | 19 | (1.33) | 1.09 | (0.69- 1.73) |
| Congenital malformation | 2,830 | (6.20) | 178 | (5.82) | 0.94 | (0.80- 1.10) | 8 | (9.52) | 1.66 | (0.77- 3.59) | 104 | (7.30) | 1.12 | (0.91- 1.38) |
| Fetal distress | 2,213 | (4.85) | 167 | (5.46) | 1.13 | (0.95- 1.33) | 10 | (11.90) | 2.79 | (1.43- 5.46) | 74 | (5.20) | 1.07 | (0.84- 1.37) |
| Cesarean delivery | 17,414 | (38.14) | 1,235 | (40.37) | 1.13 | (1.04- 1.23) | 48 | (57.14) | 2.01 | (1.32- 3.09) | 621 | (43.61) | 1.26 | (1.12- 1.43) |

Supplement table 4 Adverse birth outcomes in different anticancer treatment groups (diagnosed at 15-49 years) and the comparison group

* Numbers <3 are not displayed, as per the confidentiality policies of National Health Insurance Database

^a^ Adjusted for maternal age at delivery, infant sex, infant birth year, maternal cardiovascular disease, maternal autoimmune disease, maternal liver disease, maternal hypertension, maternal diabetes mellitus before pregnancy, maternal gestational diabetes mellitus, maternal bipolar disorder, maternal alcohol misuse during pregnancy, maternal smoking during pregnancy, maternal selective serotonin reuptake inhibitor use during pregnancy, maternal category D and X medication use during pregnancy, maternal nationality, maternal place of residence, maternal income level, maternal occupation

^b^ Cesarean delivery excluded

Supplement table 5 Adverse birth outcomes in different anticancer treatment groups in thyroid cancer survivor group and the comparison group

|  | Comparison group  N=45,463 | | Thyroid cancer survivor group | | | | | | | | | | | |
| --- | --- | --- | --- | --- | --- | --- | --- | --- | --- | --- | --- | --- | --- | --- |
|  |  |  | Neither RT nor chemotherapy  N=1,550 | | Adjusted odds ratio^a^  (95% CI) | | RT alone  N=13 | | Adjusted odds ratio^a^ (95% CI) | | Chemotherapy ± RT  N=21 | | Adjusted odds ratio^a^ (95% CI) | |
|  | N | % | N | % |  |  | N | % |  |  | N | % |  |  |
| Overall adverse birth outcome^b^ | 15,436 | (33.95) | 531 | (34.26) | 1.01 | (0.90- 1.13) | 7 | (53.85) | 2.2 | (0.79- 6.11) | 10 | (47.62) | 1.73 | (0.64- 4.68) |
| Stillbirth | 391 | (0.86) | 16 | (1.03) | 0.98 | (0.66- 1.46) | 0 | (0) | NA | | 0 | (0) | 1.16 | NA |
| Low birth weight | 3,121 | (6.86) | 105 | (6.77) | 0.97 | (0.79- 1.20) | 4 | (30.77) | 6.25 | (1.87- 20.91) | 6 | (28.57) | 5.38 | (1.70- 17.05) |
| Preterm labor | 3,788 | (8.33) | 127 | (8.19) | 0.93 | (0.77- 1.14) | 4 | (30.77) | 4.77 | (1.33- 17.12) | 3 | (14.29) | 1.55 | (0.46- 5.28) |
| Small for gestational age | 4,109 | (9.04) | 142 | (9.16) | 1.04 | (0.86- 1.24) | -* |  | 2.11 | (0.50- 8.93) | 6 | (28.57) | 4.31 | (1.41- 13.18) |
| Large for gestational age | 4,965 | (10.92) | 162 | (10.45) | 0.95 | (0.79- 1.13) | -* |  | 0.61 | (0.08- 4.81) | -* |  | 0.77 | (0.18- 3.25) |
| 5-minute Apgar Score of < 7 | 480 | (1.06) | 14 | (0.90) | 0.84 | (0.49- 1.44) | -* |  | 7.44 | (0.93- 59.70) | 0 | (0) | NA | |
| Congenital malformation | 2,817 | (6.20) | 95 | (6.13) | 0.97 | (0.78- 1.21) | -* |  | 2.72 | (0.66- 11.28) | -* |  | 0.8 | (0.10- 6.07) |
| Fetal distress | 2,205 | (4.85) | 74 | (4.77) | 0.97 | (0.76- 1.25) | 3 | (23.08) | 6.38 | (1.74- 23.37) | 3 | (14.29) | 3.22 | (0.96- 10.80) |
| Cesarean delivery | 17,297 | (38.05) | 621 | (40.06) | 1 | (0.99- 1.25) | 10 | (76.92) | 4.72 | (1.43- 15.60) | 8 | (38.10) | 1.01 | (0.36- 2.80) |

* Numbers <3 are not displayed, as per the confidentiality policies of National Health Insurance Database

^a^ Adjusted for maternal age at delivery, infant sex, infant birth year, maternal cardiovascular disease, maternal autoimmune disease, maternal liver disease, maternal hypertension, maternal diabetes mellitus before pregnancy, maternal gestational diabetes mellitus, maternal bipolar disorder, maternal alcohol misuse during pregnancy, maternal smoking during pregnancy, maternal selective serotonin reuptake inhibitor use during pregnancy, maternal category D and X medication use during pregnancy, maternal nationality, maternal place of residence, maternal income level, maternal occupation

^b^ Cesarean delivery excluded

Supplement table 6 Adverse birth outcomes in different anticancer treatment groups in gynecologic cancer survivor group and the comparison group

|  | Comparison group  N=45,463 | | Gynecologic cancer survivor group | | | | | | | | | | | |
| --- | --- | --- | --- | --- | --- | --- | --- | --- | --- | --- | --- | --- | --- | --- |
|  |  |  | Neither RT nor chemotherapy  N=485 | | Adjusted odds ratio^a^  (95% CI) | | RT alone  N=19 | | Adjusted odds ratio^a^ (95% CI) | | Chemotherapy ± RT  N=221 | | Adjusted odds ratio^a^ (95% CI) | |
|  | N | % | N | % |  |  | N | % |  |  | N | % |  |  |
| Overall adverse birth outcome^b^ | 15,436 | (33.95) | 171 | (35.26) | 1.07 | (0.88- 1.30) | 11 | (57.89) | 2.6 | (1.00- 6.74) | 83 | (37.56) | 1.21 | (0.90- 1.62) |
| Stillbirth | 391 | (0.86) | 4 | (0.82) | 0.97 | (0.36- 2.62) | 0 | (0) | NA | | -* |  | 1.18 | (0.29- 4.78) |
| Low birth weight | 3,121 | (6.86) | 49 | (10.10) | 1.54 | (1.12- 2.12) | 4 | (21.05) | 3.48 | (1.05- 11.52) | 15 | (6.79) | 0.97 | (0.56- 1.68) |
| Preterm labor | 3,788 | (8.33) | 54 | (11.13) | 1.36 | (1.01- 1.83) | 5 | (26.32) | 3.67 | (0.88- 15.35) | 25 | (11.31) | 1.46 | (0.92- 2.32) |
| Small for gestational age | 4,109 | (9.04) | 56 | (11.55) | 1.28 | (0.94- 1.73) | -* |  | 0.66 | (0.09- 4.93) | 22 | (9.95) | 1.03 | (0.65- 1.62) |
| Large for gestational age | 4,965 | (10.92) | 46 | (9.48) | 0.89 | (0.66- 1.21) | -* |  | 0.99 | (0.23- 4.22) | 26 | (11.76) | 1.25 | (0.79- 1.98) |
| 5-minute Apgar Score of < 7 | 480 | (1.06) | 10 | (2.06) | 2.04 | (1.09- 3.84) | -* |  | 5.64 | (0.97- 32.86) | 4 | (1.81) | 1.77 | (0.64- 4.87) |
| Congenital malformation | 2,817 | (6.20) | 23 | (4.74) | 0.76 | (0.49- 1.17) | -* |  | 1.77 | (0.46- 6.79) | 14 | (6.33) | 1.03 | (0.60- 1.77) |
| Fetal distress | 2,205 | (4.85) | 27 | (5.57) | 1.12 | (0.75- 1.67) | 3 | (15.79) | 3.82 | (1.08- 13.50) | 9 | (4.07) | 0.82 | (0.42- 1.57) |
| Cesarean delivery | 17,297 | (38.05) | 202 | (41.65) | 1 | (1.01- 1.53) | 11 | (57.89) | 1.87 | (0.83- 4.19) | 90 | (40.72) | 1.2 | (0.88- 1.63) |

* Numbers <3 are not displayed, as per the confidentiality policies of National Health Insurance Database

^a^ Adjusted for maternal age at delivery, infant sex, infant birth year, maternal cardiovascular disease, maternal autoimmune disease, maternal liver disease, maternal hypertension, maternal diabetes mellitus before pregnancy, maternal gestational diabetes mellitus, maternal bipolar disorder, maternal alcohol misuse during pregnancy, maternal smoking during pregnancy, maternal selective serotonin reuptake inhibitor use during pregnancy, maternal category D and X medication use during pregnancy, maternal nationality, maternal place of residence, maternal income level, maternal occupation

^b^ Cesarean delivery excluded

Supplement table 7 Adverse birth outcomes in different anticancer treatment groups in breast cancer survivor group and the comparison group

|  | Comparison group  N=45,463 | | Breast cancer survivor group | | | | | | | | | | | |
| --- | --- | --- | --- | --- | --- | --- | --- | --- | --- | --- | --- | --- | --- | --- |
|  |  |  | Neither RT nor chemotherapy  N=159 | | Adjusted odds ratio^a^  (95% CI) | | RT alone  N=15 | | Adjusted odds ratio^a^ (95% CI) | | Chemotherapy ± RT  N=517 | | Adjusted odds ratio^a^ (95% CI) | |
|  | N | % | N | % |  |  | N | % |  |  | N | % |  |  |
| Overall adverse birth outcome^b^ | 15,436 | (33.95) | 65 | (40.88) | 1.33 | (0.96- 1.83) | 8 | (53.33) | 2.31 | (0.75- 7.09) | 193 | (37.33) | 1.11 | (0.92- 1.33) |
| Stillbirth | 391 | (0.86) | -* |  | 0.62 | (0.09- 4.50) | 0 | (0) | NA | | 5 | (0.97) | 0.95 | (0.39- 2.27) |
| Low birth weight | 3,121 | (6.86) | 12 | (7.55) | 1.09 | (0.60- 1.98) | -* |  | 0.96 | (0.15- 6.01) | 31 | (6.00) | 0.78 | (0.54- 1.13) |
| Preterm labor | 3,788 | (8.33) | 15 | (9.43) | 1.04 | (0.60- 1.80) | -* |  | 0.73 | (0.14- 3.90) | 42 | (8.12) | 0.88 | (0.64- 1.22) |
| Small for gestational age | 4,109 | (9.04) | 14 | (8.81) | 1.09 | (0.63- 1.90) | -* |  | 1.75 | (0.41- 7.48) | 34 | (6.58) | 0.74 | (0.52- 1.06) |
| Large for gestational age | 4,965 | (10.92) | 23 | (14.47) | 1.25 | (0.76- 2.07) | -* |  | 0.58 | (0.08- 4.54) | 78 | (15.09) | 1.34 | (1.04- 1.72) |
| 5-minute Apgar Score of < 7 | 480 | (1.06) | 4 | (2.52) | 2.24 | (0.81- 6.21) | 0 | (0) | NA | | -* |  | 1.01 | (0.47- 2.15) |
| Congenital malformation | 2,817 | (6.20) | 10 | (6.29) | 1.08 | (0.57- 2.04) | 4 | (26.67) | 5.74 | (1.69- 19.49) | 44 | (8.51) | 1.27 | (0.92- 1.77) |
| Fetal distress | 2,205 | (4.85) | 19 | (11.95) | 2.74 | (1.71- 4.39) | -* |  | 1.5 | (0.19- 11.61) | 31 | (6.00) | 1.25 | (0.86- 1.82) |
| Cesarean delivery | 17,297 | (38.05) | 62 | (38.99) | 1 | (0.71- 1.46) | 11 | (73.33) | 3.29 | (1.26- 8.59) | 218 | (42.17) | 1.11 | (0.91- 1.34) |

* Numbers <3 are not displayed, as per the confidentiality policies of National Health Insurance Database

^a^ Adjusted for maternal age at delivery, infant sex, infant birth year, maternal cardiovascular disease, maternal autoimmune disease, maternal liver disease, maternal hypertension, maternal diabetes mellitus before pregnancy, maternal gestational diabetes mellitus, maternal bipolar disorder, maternal alcohol misuse during pregnancy, maternal smoking during pregnancy, maternal selective serotonin reuptake inhibitor use during pregnancy, maternal category D and X medication use during pregnancy, maternal nationality, maternal place of residence, maternal income level, maternal occupation

^b^ Cesarean delivery excluded

Supplement table 8 Adverse birth outcomes in different anticancer treatment groups in cancer survivor group with age of cancer diagnosis (<30 and ≥30 years) compared to the comparison group

|  | Cancer survivor group | | | | | | | | | | | |
| --- | --- | --- | --- | --- | --- | --- | --- | --- | --- | --- | --- | --- |
|  | **Age of cancer diagnosis < 30** | | | | | | **Age of cancer diagnosis ≥ 30** | | | | | |
|  | Neither RT nor chemotherapy | | RT alone | | Chemotherapy ± RT | | Neither RT nor chemotherapy | | RT alone | | Chemotherapy ± RT | |
|  | (N=2,269) | | (N=54) | | (N=921) | | (N=776) | | (N=30) | | (N=497) | |
|  | Adjusted odds ratio^a^ (95% CI) | | | | | | | | | | | |
| Overall adverse birth outcome^b^ | 1.08 | (0.99- 1.19) | 2.26 | (1.28- 4.00) | 1.12 | (0.98- 1.30) | 1.01 | (0.86- 1.17) | 2.73 | (1.22- 6.12) | 1.04 | (0.86- 1.26) |
| Stillbirth | 0.97 | (0.61- 1.56) | NA |  | 0.97 | (0.46- 2.03) | 0.98 | (0.49- 1.99) | NA |  | 1.42 | (0.71- 2.87) |
| Low birth weight | 1.09 | (0.92- 1.30) | 2.58 | (1.24- 5.39) | 1.27 | (0.99- 1.63) | 1.06 | (0.81- 1.40) | 1.76 | (0.53- 5.86) | 1.17 | (0.87- 1.59) |
| Preterm labor | 1.11 | (0.95- 1.30) | 2.36 | (1.13- 4.92) | 1.33 | (1.06- 1.66) | 0.96 | (0.75- 1.24) | 2.45 | (0.79- 7.58) | 0.94 | (0.69- 1.28) |
| Small for gestational age | 1.05 | (0.90- 1.22) | 2.16 | (1.06- 4.40) | 1 | (0.79- 1.26) | 1.25 | (0.98- 1.59) | 0.47 | (0.06- 3.39) | 0.96 | (0.69- 1.34) |
| Large for gestational age | 1 | (0.86- 1.16) | 1.12 | (0.48- 2.65) | 1.05 | (0.83- 1.32) | 1.04 | (0.83- 1.29) | 1.66 | (0.62- 4.46) | 1.09 | (0.84- 1.42) |
| 5-minute Apgar Score of < 7 | 1.06 | (0.68- 1.65) | 2.09 | (0.29- 15.20) | 1.08 | (0.58- 2.02) | 1.17 | (0.65- 2.10) | 6.07 | (1.67- 22.14) | 1.19 | (0.60- 2.33) |
| Congenital malformation | 1 | (0.83- 1.20) | 1.66 | (0.60- 4.56) | 1.06 | (0.81- 1.39) | 0.77 | (0.56- 1.08) | 1.67 | (0.51- 5.45) | 1.24 | (0.89- 1.72) |
| Fetal distress | 1.18 | (0.97- 1.42) | 2.99 | (1.33- 6.73) | 0.99 | (0.72- 1.35) | 0.99 | (0.70- 1.38) | 2.37 | (0.71- 7.86) | 1.19 | (0.80- 1.78) |
| Cesarean delivery | 1.14 | (1.03- 1.26) | 2.08 | (1.22- 3.54) | 1.3 | (1.12- 1.52) | 1.1 | (0.94- 1.28) | 1.85 | (0.94- 3.64) | 1.18 | (0.97- 1.44) |

^a^ Adjusted for maternal age at delivery, infant sex, infant birth year, maternal cardiovascular disease, maternal autoimmune disease, maternal liver disease, maternal hypertension, maternal diabetes mellitus before pregnancy, maternal gestational diabetes mellitus, maternal bipolar disorder, maternal alcohol misuse during pregnancy, maternal smoking during pregnancy, maternal selective serotonin reuptake inhibitor use during pregnancy, maternal category D and X medication use during pregnancy, maternal nationality, maternal place of residence, maternal income level, maternal occupation

^b^ Cesarean delivery excluded

Supplement table 9 Adverse birth outcomes in different anticancer treatment groups in cancer survivor group with different length of duration between cancer diagnosis and delivery (<3 years and ≥ 3 years) compared to the comparison group

|  | Duration between cancer diagnosis and delivery <3 years | | | | | | Duration between cancer diagnosis and delivery ≥3 years | | | | | |
| --- | --- | --- | --- | --- | --- | --- | --- | --- | --- | --- | --- | --- |
|  | Neither RT nor chemotherapy | | RT alone | | Chemotherapy ± RT | | Neither RT nor chemotherapy | | RT alone | | Chemotherapy ± RT | |
|  | (N=1289) | | (N=40) | | (N=652) | | (N=1756) | | (N=44) | | (N=766) | |
|  | Adjusted odds ratio^a^ (95% CI) | | | | | | | | | | | |
| Overall adverse birth outcome^b^ | 1.02 | (0.91- 1.15) | 4.54 | (2.24- 9.22) | 1.06 | (0.90- 1.24) | 1.09 | (0.98- 1.21) | 1.35 | (0.71- 2.55) | 1.13 | (0.97- 1.31) |
| Stillbirth | 1.22 | (0.70- 2.12) | NA |  | 1.72 | (0.92- 3.22) | 0.82 | (0.47- 1.43) | NA |  | 0.71 | (0.30- 1.71) |
| Low birth weight | 1.07 | (0.86- 1.34) | 2.07 | (0.82- 5.22) | 1.27 | (0.96- 1.67) | 1.08 | (0.89- 1.30) | 2.25 | (0.97- 5.25) | 1.19 | (0.91- 1.55) |
| Preterm labor | 1.02 | (0.83- 1.25) | 2.39 | (0.91- 6.27) | 1.06 | (0.81- 1.39) | 1.1 | (0.93- 1.31) | 2.37 | (1.05- 5.33) | 1.25 | (0.98- 1.59) |
| Small for gestational age | 1.12 | (0.93- 1.35) | 1.58 | (0.63- 3.97) | 0.98 | (0.74- 1.28) | 1.08 | (0.91- 1.28) | 1.46 | (0.58- 3.66) | 0.99 | (0.76- 1.28) |
| Large for gestational age | 0.97 | (0.81- 1.16) | 1.75 | (0.72- 4.25) | 1.17 | (0.92- 1.49) | 0.94 | (0.77- 1.16) | 1.57 | (0.49- 5.02) | 1.06 | (0.80- 1.41) |
| 5-minute Apgar Score of < 7 | 1.23 | (0.74- 2.04) | 7.51 | (2.58- 21.90) | 1.2 | (0.62- 2.33) | 0.99 | (0.61- 1.61) | NA |  | 1.07 | (0.57- 2.01) |
| Congenital malformation | 0.93 | (0.73- 1.19) | 1.71 | (0.62- 4.71) | 1.21 | (0.90- 1.63) | 1.04 | (0.89- 1.22) | 0.97 | (0.38- 2.45) | 0.98 | (0.78- 1.25) |
| Fetal distress | 1.09 | (0.85- 1.39) | 2.89 | (1.10- 7.55) | 0.85 | (0.57- 1.25) | 1.15 | (0.93- 1.43) | 2.68 | (1.04- 6.89) | 1.27 | (0.93- 1.72) |
| Cesarean delivery | 1.05 | (0.94- 1.19) | 1.28 | (0.70- 2.35) | 1.27 | (1.07- 1.49) | 1.16 | (1.04- 1.29) | 2.91 | (1.60- 5.29) | 1.23 | (1.04- 1.44) |

^a^ Adjusted for maternal age at delivery, infant sex, infant birth year, maternal cardiovascular disease, maternal autoimmune disease, maternal liver disease, maternal hypertension, maternal diabetes mellitus before pregnancy, maternal gestational diabetes mellitus, maternal bipolar disorder, maternal alcohol misuse during pregnancy, maternal smoking during pregnancy, maternal selective serotonin reuptake inhibitor use during pregnancy, maternal category D and X medication use during pregnancy, maternal nationality, maternal place of residence, maternal income level, maternal occupation

^b^ Cesarean delivery excluded
